# The Relationship between Fragmented QRS and Myocardial Injury in Patients with Acute Carbon Monoxide Poisoning

**DOI:** 10.3390/medicina60060891

**Published:** 2024-05-28

**Authors:** Osman Küçükkelepçe, Emre Yılmaz, Sencer Çamcı

**Affiliations:** 1 Department of Public Health, Adıyaman Provincial Health Directorate, 02100 Adıyaman, Turkey; 2 Department of Cardiology, Faculty of Medicine, Giresun University, 28100 Giresun, Turkey; dremreyilmaz@hotmail.com (E.Y.); sncrcmc@gmail.com (S.Ç.)

**Keywords:** carbon monoxide poisoning, electrocardiography, fragmented QRS, myocardial injury

## Abstract

*Background and Objectives:* Carbon monoxide (CO) intoxication is one of the most common causes of poisoning-related deaths and complications. Myocardial injury is an important complication of CO poisoning. In our study, we aimed to evaluate the relationship between the presence and prevalence of fragmented QRS (fQRS) and myocardial injury in patients with CO intoxication. *Materials and Methods:* We retrospectively evaluated patients who presented to the emergency department of our tertiary care center with CO intoxication between January 2020 and December 2023. In our study, we performed subgroup analyses according to the presence of myocardial injury and fQRS. We evaluated the parameters and risk factors associated with myocardial injury. *Results:* Myocardial injury was detected in 44 patients, and fQRS was detected in 38 patients. In the myocardial injury (+) group, the fQRS rate was 38.6%, and the median number of leads with fQRS was 3 (2–6) and was significantly higher than in the myocardial injury (−) group (*p* < 0.001). We found that carboxyhemoglobin had a significant positive correlation with troponin (*p* = 0.001) and pro-B-type natriuretic peptide (proBNP) (*p* = 0.009). As a result of multivariate analysis, we determined that age, creatinine, proBNP, fQRS, and ≥3 leads with fQRS are independent risk factors for myocardial injury. *Conclusions:* Myocardial injury in CO intoxication patients is associated with proBNP, the presence of fQRS, and the number of leads with fQRS. Age, creatinine level, proBNP, the presence of fQRS, and ≥3 leads with fQRS are independent risk factors for myocardial injury in patients with CO intoxication.

## 1. Introduction

Carbon monoxide (CO) is a colorless, tasteless, odorless, and non-irritating gas that can be found in trace amounts in the air we breathe and in toxic doses in the smoke resulting from the incomplete combustion of carbon-containing fuels [1,2]. CO poisoning is one of the most common causes of poisoning-related deaths and complications [3]. The clinical presentation of patients with CO poisoning varies widely and is influenced by the duration of exposure and the CO concentration. Patients may experience mild to moderate symptoms such as headaches, weakness, or dizziness, but severe clinical effects such as convulsions, cardiovascular collapse, or even death can also occur [4,5]. Myocardial injury is an important complication of CO poisoning. CO poisoning reduces the oxygen-carrying capacity of the blood. It has been suggested that the most likely underlying cause of myocardial injury after CO poisoning is carboxyhemoglobin (COHb)-induced hypoxia. This can cause some electrical, morphological, and functional changes in the heart [6,7]. The degree of cardiotoxicity in patients with CO intoxication is highly variable. With CO, the ventricular fibrillation threshold may decrease, and electrocardiographic changes such as ST-segment depression, ischemia, atrial flutter, atrial fibrillation, premature ventricular tachycardia, and conduction system disorders may also be observed [8,9].

Arrhythmogenic susceptibility and myocardial dysfunction caused by CO intoxication have been evaluated in studies with different designs. Ventricular repolarization markers such as QTc, the T_peak_-T_end_ interval, T_peak_-T_end_ dispersion, QT dispersion, T_peak_-T_end_/QT, and T_peak_-T_end_/QTc were evaluated in patients followed up due to CO intoxication compared to healthy control group individuals [10,11,12,13]. Ventricular repolarization markers have also been shown to be associated with myocardial injury in patients with CO intoxication [12]. It has been reported that mortality and morbidity were observed in some cases even if CO and cardiac biomarker levels improved during and after CO intoxication treatment. At this stage, it was thought that an unstable pathology such as myocardial fibrosis may underlie arrhythmogenic predisposition and poor cardiac outcomes. For this purpose, cardiac fibrosis in CO intoxication patients was evaluated with cardiac magnetic resonance imaging, and late gadolinium retention and fibrosis were found to be higher in the patient group with myocardial injury [14]. 

Fragmented QRS (fQRS) is a low-cost test that can be easily detected from a 12-lead surface electrocardiogram (ECG) and is a depolarization disorder due to conduction delay caused by myocardial fibrosis [15]. fQRS has been associated with an increased risk of cardiovascular mortality and morbidity in patients with acute coronary syndrome, pulmonary embolism, and hypertension [16,17,18]. However, there is not enough information in the literature to evaluate an easily accessible parameter such as fQRS, which is associated with cardiovascular mortality and morbidity in CO intoxication patients. In our study, we aimed to evaluate the relationship between the presence and prevalence of fQRS and myocardial injury in patients with CO intoxication.

## 2. Materials and Methods

In our study, we evaluated CO intoxication patients who visited the emergency department of our tertiary care center between January 2020 and December 2023. Our study was conducted with a single-center, cross-sectional, observational, and retrospective design, in accordance with the principles of the Declaration of Helsinki, with the approval of the ethics committee of Giresun Training and Research Hospital (approval date and number: 06.03.2024/01)

Patients with carbon monoxide poisoning who were treated at the center mentioned above between 2020 and 2023 were included in the study. These patients were recorded in the hospital information system under the ICD-10 diagnosis code T58 for carbon monoxide toxic effect. Their records were examined retrospectively. The prerequisite for patient acceptance in our study was that the patient was ≥18 years old and that the COHb rate was ≥10%, in accordance with other examples in the literature [12,19]. Our other inclusion criteria were a body/mass index of 20–30 kg/m^2^ and the presence of an ECG record in the file scan that could be transferred to electronic media.

Our exclusion criteria were the presence of coronary artery disease, diabetes mellitus, hypertension, hypertrophic cardiomyopathy, known electrolyte disorder, advanced valve disease, heart failure with reduced ejection fraction, a pacemaker, advanced renal and liver failure, and antiarrhythmic drug use. As a result, we included 190 patients in our study.

We accessed patients’ demographic data and medical histories through file scans and health databases. For CO intoxication, we obtained data on the duration of exposure, the season in which the intoxication event occurred, and the region where the intoxication victim resided.

At our center, CO intoxication patients are evaluated in the red area of our emergency department. In this unit, arterial blood gas, hemogram, biochemistry analyses, and ECG measurements are routinely performed for each patient. For this reason, we evaluated the blood gas, hemogram data, biochemistry analysis results, and ECG measurements of our study patients in the analysis. Since echocardiographic measurements are not routinely performed for every patient presenting with CO intoxication, we did not include them in the analysis. Additionally, since there is no hyperbaric oxygen therapy unit in our center, patients needing this therapy were transferred to different centers. It was not possible for us to access the follow-ups and data regarding the hospitalization or discharge process for every patient. For this reason, we analyzed the data obtained during the application process.

In our study, we performed subgroup analyses according to the presence of myocardial injury and fQRS. The presence of myocardial injury was defined by a measurement of the troponin level above the 99th percentile of the reference accepted for the healthy population. Troponin T is used in our center, and we defined the upper limit of 0.014 µg/L and above as a high troponin value and the presence of myocardial injury [12]. With this grouping, we categorized patients with a troponin T value ≥ 0.014 µg/L in the ‘myocardial injury (+)’ group and patients with a troponin T value < 0.014 µg/L in the ‘myocardial injury (−)’ group. Adhering to the original definition of Das et al. [20], we categorized patients with fQRS on ECG in the ‘fQRS (+)’ group and patients without fQRS in the ‘fQRS (−)’ group.

### 2.1. Laboratory Analysis Details

We evaluated arterial blood gas, hemogram, biochemistry, and cardiac biomarkers measured during the patients’ emergency admissions in our analyses. CO was assessed by measuring the blood COHb level from arterial blood using the Cobas b 221 Blood Gas System (Roche Diagnostics, Inc., Indianapolis, IN, USA). Complete blood counts were evaluated with a Beckman Coulter LH 780 hematology analyzer. Biochemical parameters were assessed using Cobas 6000 (Roche, Basel, Switzerland). The laboratory measurements of the patients were from the period of their first admission and were studied in our central biochemistry laboratory. We obtained the measurement results through archive scans and health databases.

### 2.2. Electrocardiography Measurement Details

The baseline ECGs of our study patients (Cardiofax GEM, model 9022 K, Nihon Kohden, Tokyo, Japan) were recorded in the supine position during admission to the emergency department, standardized at a speed of 25 mm/s and an amplitude of 10 mm/mV. We evaluated heart rate (HR), the PR interval, the QT interval, QT corrected by the Bazett formula (QTc), the presence of fQRS, and the number of leads with fQRS in our analyses. We obtained our measurements manually from each lead of a 12-lead ECG and took their average. We also adopted the fact that at least 8 leads in a 12-lead ECG were suitable for measurements as a prerequisite for inclusion. If the measurement quality was low in any lead, we took the average of the measurements of 3 consecutive beats of the same lead. We measured the PR interval as the time from the beginning of the P wave to the beginning of the QRS complex. We measured the QT interval as the time from the beginning of the Q wave to the end of the T wave. QTc was calculated with the Bazett formula: QTc = QT/RR^1/2^.

**Figure 1:**
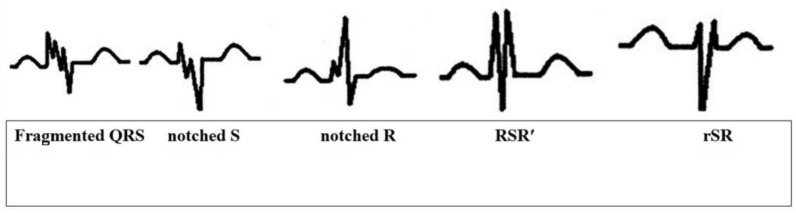
Different presentations of fQRS on ECG.

Fragmented QRS, as in its original definition and other case studies in the literature, is the presence of an additional R wave (R’), indicating a major coronary artery territory, observed in at least 2 consecutive leads, or QRS accompanied by a notching in the R wave or the descending limb of the S-wave complex or more than one R’ (fragmentation) (Figure 1) [20].

We evaluated the ECGs we obtained from electronic archive scanning using magnifiers and measurement tools. Intra- and interobserver coefficients of variation (the standard deviation of differences between two observations divided by the mean value and expressed as a percentage) were found to be less than 4% for all measurements. ECGs in the study group were measured by an individual blinded to clinical exposure by the authors SÇ and EY.

### 2.3. Statistical Analysis

Statistical analyses were conducted using SPSS version 22 (SPSS/IBM, IBM Corp., Armonk, NY, USA). Numerical variables are presented as mean [standard deviation (SD)], while categorical variables are expressed as number (*n*) and percentage (%). The normal distribution of the data was assessed using the Kolmogorov–Smirnov test. The Student’s *t*-test was employed to compare normally distributed groups, and the Mann–Whitney U-test was utilized for non-normally distributed groups. Qualitative variables between groups were compared using the Chi-square test. Pearson correlation analysis was employed to evaluate relationships between numerical variables. Logistic regression analysis was carried out to identify independent predictors of myocardial injury. All hypotheses were two-sided, and statistical significance was set at *p*-value < 0.05.

## 3. Results

The mean age of the 190 patients included in our study was 42.05 ± 18.16 years (minimum of 18 and maximum of 81), and the male sex ratio was 36.3% (*n* = 69). We found that 66% of our CO intoxication cases occurred in the winter months and 51% in rural subjects. We present the general characteristics of our study group in Table 1.

Our prerequisite was that our study patients’ COHb levels were above 10%. For this reason, we determined the average COHb level of our study group to be 17.82 (3.42)%. There were no features in the arterial blood gas measurements of our study patients other than the lactate level. We found the mean lactate level to be 2.46 (0.48) mmol/L. There were no abnormalities in the electrolyte levels or other biochemical measurements of our study patients. However, the mean levels of troponin T [0.028 (0.005) µg/L] and pro-B-type natriuretic peptide (proBNP) [257.64 (50.52) pg/mL] were above the reference values. We present the laboratory test results of our study group in Table 2.

We present the subgroup analysis results of our study group in Table 3. The mean age of the myocardial injury (+) subgroup was higher than that of the myocardial injury (−) subgroup. We found that COHb, lactate, creatinine, creatine kinase (CK), and proBNP levels were significantly higher in the myocardial injury (+) group. When ECG measurements were compared, we observed that the heart rate of the myocardial injury (+) group was higher. We found that the presence of fQRS was higher in the same group, and the number of leads with fQRS was higher. In our subgroup analysis based on the presence of fQRS, we observed that the age of the fQRS (+) group was older and the male sex ratio was higher. Creatinine, troponin T, and proBNP levels were significantly lower in the fQRS (−) group. We found that PR and QTc measurements were higher in the fQRS (+) group.

In the correlation analysis, we evaluated the interrelationships of variables with different designs. First of all, we found that COHb had significant positive correlations with troponin T (*r* = 0.582; *p* = 0.001) and proBNP (*r* = 0.468; *p* = 0.009). We found that troponin T had significant positive correlations with age (*r* = 0.281; *p* = 0.002), lactate (*r* = 0.203; *p* = 0.01), creatinine (*r* = 0.217; *p* = 0.009), proBNP (*r* = 0.598; *p* < 0.001), fQRS (*r* = 0.569; *p* = 0.001), and the number of leads with fQRS (*r* = 0.494; *p* = 0.005) in our study group. Finally, we found that fQRS had significant positive correlations with age (*r* = 0.204; *p* = 0.03), creatinine (*r* = 0.221; *p* = 0.02), troponin T (*r* = 0.406; *p* = 0.001), and proBNP (*r* = 0.361; *p* = 0.002).

We applied multivariate logistic regression analysis to identify risk factors for myocardial injury in our study population. In modeling, we used the variables age, COHb, lactate, creatinine, proBNP, fQRS, and number of leads with fQRS, which had significant odds ratios (ORs) in the univariate analysis. As a result of the multivariate analysis, we found that age [OR: 1.089; 95% confidence interval (CI): 1.011–1.162; *p* = 0.02], creatinine (OR: 1.097; 95% CI: 1.009–1.305; *p* = 0.009), proBNP (OR: 1.836; %95 CI: 1.256–2.588; *p* = 0.001), and fQRS (OR: 2.187; 95% CI: 1.809–3.401; *p* < 0.001) variables were independent risk factors for myocardial injury. In further modeling, we found that ≥3 leads with fQRS (OR: 2.398; 95% CI: 1.744–4.108; *p* < 0.001) was an independent risk factor for myocardial injury (Table 4).

## 4. Discussion

In this study, we evaluated the relationship between the presence and prevalence of fQRS and myocardial injury in patients with CO intoxication. We performed subgroup analyses according to the presence of myocardial injury and fQRS. We evaluated the parameters and risk factors associated with myocardial injury. fQRS was observed more frequently in patients with myocardial injury. In addition, proBNP and fQRS leads were also detected as indicators of myocardial injury. Age, creatinine level, proBNP, the presence of fQRS, and ≥3 leads with fQRS were found to be independent risk factors for myocardial injury in patients with CO intoxication.

CO poisoning, which is thought to be responsible for more than half of the deaths due to poisoning worldwide, is a serious health problem with high morbidity and mortality rates [21]. CO poisoning reduces the oxygen-carrying capacity of the blood. Additionally, CO binds to myocardial myoglobin and reduces myocardial oxygen reserve [22]. As a result, myocardial injury occurs in a significant proportion of CO intoxication patients [8,9]. As a result of this injury, atrial fibrillation, ventricular extrasystole, ventricular tachycardia/fibrillation, atrioventricular block, acute myocardial infarction, and acute heart failure may occur in patients [8,9,23]. Death from cardiovascular causes was significantly higher in patients with myocardial injury [24]. Therefore, assessing myocardial injury and its severity as early as possible is of high importance for guiding its treatment. Levels of cardiac markers increase due to myocardial injury caused by CO poisoning. Measurements of troponin, BNP, CK, and CK-myocardial band levels in patients with CO poisoning help to determine the degree of cardiac injury or dysfunction [6,24,25]. In our study, we used troponin T to determine myocardial injury groups.

Fragmented QRS is defined as various RSR’ patterns [20]. It is accepted that QRS complex fragmentation is caused by regional myocardial fibrosis/scar and ischemia leading to heterogeneous myocardial electrical activation and unsynchronized contraction [26]. The occurrence of fQRS in acute coronary syndrome patients predicts the occurrence of heart failure [27]. fQRS was found to be associated with myocardial fibrosis in patients with ischemic or non-ischemic left ventricular dysfunction and predicted increased morbidity and mortality [27,28]. In addition to the presence of fQRS, the prognostic value of a number ≥2 has also been investigated in studies. It has been found to significantly predict mortality in acute coronary syndrome patients [29]. A greater number of fQRS leads has been shown to be associated with worse left ventricular function, higher troponin levels, and lower ST resolution rates [30,31,32]. In the study by Tanrıverdi et al. [33] in acute ST-segment elevation myocardial infarction patients, the in-hospital mortality rate was significantly higher in patients with fQRS ≥ 3 compared with patients with fQRS < 3. Similarly, Yildirim et al. [34] found that major adverse cardiac events increased significantly as the number of fQRS leads increased, especially in acute MI patients with ≥4 fQRS leads. Torigoe et al. [35] observed similar findings in patients with prior MI. They found that the number of leads with fQRS was an independent predictor of cardiac death or hospitalization for heart failure. In patients with idiopathic dilated cardiomyopathy, both the number of leads with fQRS and the duration of the fQRS complex have been shown to have an inverse relationship with left ventricular ejection fraction [36,37]. In our study, in accordance with the previous discussion, the presence of fQRS and the number of leads with fQRS ≥ 3 were found to be independent risk factors for myocardial injury in patients with CO intoxication.

BNP is a hormone with local cytoprotective and antiproliferative effects and systemic vasodilator effects and is released mainly from the left ventricle and, to a lesser extent, from the atrial myocardium and brain [38]. It is known that myocardial hypoxia contributes to increased myocardial wall stretching and hypoperfusion, which leads to an increase in BNP production by stimulating stretch receptors [39]. In patients with myocardial injury, an increase in N-terminal-proBNP levels has been found to indicate myocardial injury despite the absence of electrocardiographic and echocardiographic changes [40]. Kalay et al. [6] found that BNP levels were negatively correlated with left ventricular function. In our study, we analyzed proBNP levels and found significantly higher levels in the myocardial injury group.

With advancing age, more ischemic events occur in the heart [41]. Atherosclerotic burden increases with age [42]. Endothelium-mediated vasodilatation decreases with decreased nitric oxide. This limits maximum coronary blood flow in response to increased myocardial oxygen demand [43]. Aging is also associated with increased left ventricular afterload due to increased arteriosclerosis and increased preload due to increased myocardial stiffness [43,44]. These changes contribute to an imbalance between myocardial oxygen supply and demand, which exacerbates ischemia. There is also an increased tendency to develop thrombosis with increasing age [45]. With the contribution of these and similar factors, the myocardium becomes more prone to ischemia. Similarly, the mean age of the myocardial injury group was higher in our study.

In our study, the creatinine level was also found to be an independent risk factor for cardiac injury in CO intoxication patients. Numerous studies have shown that elevated serum creatinine may be an independent predictor of all-cause and cardiovascular disease mortality [46,47]. Increased creatinine was found to be a predictor of increased cardiovascular risk in hypertensive patients [48]. In patients with acute myocardial infarction, high creatinine levels on admission were found to be an independent risk factor for increased mortality [49]. 

It is important to recognize the presence of myocardial injury in CO intoxication patients with high cardiac morbidity and mortality in order to start treatment rapidly. The presence of fQRS on ECG has been established as a marker of cardiac injury and dysfunction in many previous studies [27,50]. To the best of our knowledge, our study, which is the first in the literature, has shown that the presence of fQRS on ECG in CO intoxication patients is a candidate to enter our clinical practice as a strong predictor to identify patients with myocardial injury.

Our study has some limitations. Our first limitation is that the number of participants is low. However, in accordance with our sample size analysis, we tried to reach the highest possible number in line with the examples in the literature. Another limitation of our study is its retrospective design. We primarily chose this method to test the validity of our hypothesis and lead to prospective designs. On the other hand, our retrospective design prevented us from accessing the patients’ echocardiography data and post-discharge data. Finally, the absence of a hyperbaric oxygen therapy unit in our center prevented us from accessing the in-hospital data of patients who needed this treatment. For this reason, we chose to analyze the data obtained at the time of emergency admission in our study.

## 5. Conclusions

Myocardial injury in CO intoxication patients is associated with proBNP, the presence of fQRS, and the number of leads with fQRS. Age, creatinine level, proBNP, the presence of fQRS, and ≥3 leads with fQRS are independent risk factors for myocardial injury in patients with CO intoxication.

## Figures and Tables

**Table 1:** The general characteristics of the study group.

Variables	Mean Values ± SD	%
Age (years)	42.05 ± 18.16	
Male gender (%)	69	36.3
BMI (kg/m^2^)	26.64 ± 3.21	
Duration of CO exposure (h)	4 (1–9) *	
Intox season		
Spring	38	20
Summer	11	6
Autumn	15	8
Winter	126	66
Intox area		
City	58	31
Village	97	51
Both	35	18

SD: standard deviation; BMI: body mass index; CO: carbon monoxide; * median (minimum–maximum).

**Table 2:** The laboratory characteristics of the study group.

Variables	Mean Values ± SD	Normal Range
pH	7.42 ± 0.159	7.35–7.45
pCO_2_ (mmHg)	41.53 ± 8.47	35–48
PaO_2_ (mmHg)	85.24 ± 16.71	83–108
HCO_3_ (mEq/L)	23.82 ± 3.96	22–26
BE (mEq/L)	1.4 (−1.9–2.3) *	(−2)–(+2)
SpO_2_ (%)	98.28 ± 18.54	95–100
COHb (%)	17.82 ± 3.42	<10%
Lactate (mmol/L)	2.46 ± 0.48	0.5–1.6
Sodium (mmol/L)	139.25 ± 24.62	136–145
Potassium (mmol/L)	4.22 ± 0.84	3.5–5.1
Chloride (mmol/L)	108.62 ± 19.27	98–107
Calcium (mg/dL)	9.28 ± 1.87	8.8–10.2
Hemoglobin (g/dL)	12.63 ± 2.52	12.1–17.2 **
Platelet (10^9^/L)	256.61 ± 48.41	140–450
Alanine aminotransferase (U/L)	24.46 ± 4.91	0–41
Aspartate aminotransferase (U/L)	26.12 ± 5.26	0–40
Creatinine (mg/dL)	1.09 ± 0.21	0.7–1.2
Creatine kinase (U/L)	112.68 ± 21.66	0–190
Troponin T (µg/L)	0.028 ± 0.005	0–0.014
ProBNP (pg/mL)	257.64 ± 50.52	0–125

SD: standard deviation; pCO_2_: partial carbon dioxide pressure; PaO_2_: partial arterial oxygen pressure; HCO_3_: bicarbonate; BE: base excess; SpO_2_: peripheral oxygen saturation; COHb: carboxyhemoglobin; proBNP: pro-B-type natriuretic peptide; * median (minimum–maximum); ** male: 13.8–17.2 g/dL; female: 12.1–15.1 g/dL.

**Table 3:** General characteristics and laboratory and electrocardiography measurement results of myocardial injury and fragmented QRS subgroups.

	Myocardial Injury (+) (*n* = 44)	Myocardial Injury (−) (*n* =146)	*p*-Value	Fragmented QRS (+) (*n* = 38)	Fragmented QRS (−) (*n* = 152)	*p*-Value
Age (years)	48.26 ± 11.5	40.69 ± 10.35	**0.011**	49.61 ± 13.86	39.16 ± 10.03	**<0.001**
Male gender (%)	17 (38.6%)	52 (35.6%)	0.50	16 (42.1%)	53 (34.8%)	**0.005**
COHb (%)	21.87 ± 4.36	15.24 ± 3.03	**<0.001**	19.46 ± 4.14	18.84 ± 3.62	0.10
Lactate (mmol/L)	5.08 ± 0.98	2.28 ± 0.43	**0.003**	2.68 ± 0.71	2.29 ± 0.42	0.34
Creatinine (mg/dL)	1.42 ± 0.26	1.02 ± 0.21	**<0.001**	1.39 ± 0.24	1.13 ± 0.25	**0.01**
CK (U/L)	134.77 ± 25.94	108.79 ± 21.74	**0.009**	114.08 ± 24.49	110.85 ± 22.96	0.24
Troponin T (µg/L)	0.102 ± 0.021	0.011 ± 0.002	**<0.001**	0.086 ± 0.013	0.026 ± 0.006	**0.009**
ProBNP (pg/mL)	806.21 ± 142.5	105.46 ± 20.98	**<0.001**	614.47 ± 102.9	111.63 ± 19.25	**0.001**
HR (/min)	92.85 ± 18.57	78.69 ± 15.43	**0.002**	81.47 ± 13.62	82.78 ± 14.52	0.5
PR(ms)	173.08 ± 32.61	171.82 ± 31.46	0.531	175.12 ± 35.82	170.24 ± 33.46	**0.004**
QT (ms)	360.81 ± 62.16	359.09 ± 61.81	0.16	361.24 ± 65.06	359.47 ± 63.62	0.26
QTc (ms)	435.26 ± 82.05	433.08 ± 84.61	0.41	436.72 ± 86.51	431.29 ± 84.15	**0.008**
Fragmented QRS	17 (38.6%)	21 (14.38%)	**<0.001**	38 (100%)	-	-
Number of leads with fragmented QRS	3 (2–6) *	2 (2–3) *	**<0.001**	2 (2–6) *	-	-

COHb: carboxyhemoglobin; CK: creatine kinase; proBNP: pro-B-type natriuretic peptide; HR: heart rate; * median (minimum–maximum). The bold indicates statistical significance.

**Table 4:** Multivariate logistic regression analysis to identify risk factors for myocardial injury.

	OR	95% CI	*p*-Value
Age	1.089	1.011–1.162	**0.02**
COHb	1.054	0.981–1.615	0.20
Lactate	1.039	0.962–1.208	0.14
Creatinine	1.097	1.009–1.305	**0.009**
proBNP	1.836	1.256–2.588	**0.001**
fQRS	2.187	1.809–3.401	**<0.001**
≥3 leads with fQRS	2.398	1.744–4.108	**<0.001**

OR: odds ratio; CI: confidence interval; COHb: carboxyhemoglobin; proBNP: pro-B-type natriuretic peptide; fQRS: fragmented QRS.

## Data Availability

Data will be available upon reasonable request from the corresponding author.

## References

[1] Bucak I.H., Tanrıverdi H., Kılıç F.E. (2023). An evaluation of childhood carbon monoxide intoxications in a rural area using the Beaufort wind scale. Environ. Monit. Assess..

[2] Öz E., Küçükkelepçe O., Kurt O., Vural A. (2023). Carbon monoxide poisoning: Beyond survival—Mortality, morbidities, and risk factors, a Turkey sample. PeerJ.

[3] Cobb N., Etzel R.A. (1999). Unintentional carbon monoxide-related deaths in the United States, 1979 through 1988. JAMA.

[4] Garg J., Krishnamoorthy P., Palaniswamy C., Khera S., Ahmad H., Jain D., Aronow W.S., Frishman W.H. (2018). Cardiovascular Abnormalities in Carbon Monoxide Poisoning. Am. J. Ther..

[5] Kao H.K., Lien T.C., Kou Y.R., Wang J.H. (2009). Assessment of myocardial injury in the emergency department independently predicts the short-term poor outcome in patients with severe carbon monoxide poisoning receiving mechanical ventilation and hyperbaric oxygen therapy. Pulm. Pharmacol. Ther..

[6] Kalay N., Ozdogru I., Cetinkaya Y., Eryol N.K., Dogan A., Gul I., Inanc T., Ikızceli I., Oguzhan A., Abaci A. (2007). Cardiovascular effects of carbon monoxide poisoning. Am. J. Cardiol..

[7] Baydin A., Amanvermez R., Çelebi H.E., Tuncel O.K., Demircan S. (2016). Pentraxin 3, ischemia-modified albumin, and myeloperoxidase in predicting a cardiac damage in acute carbon monoxide poisoning. Am. J. Emerg. Med..

[8] Gandini C., Castoldi A.F., Candura S.M., Locatelli C., Butera R., Priori S., Manzo L. (2001). Carbon monoxide cardiotoxicity. J. Toxicol. Clin. Toxicol..

[9] Dahms T.E., Younis L.T., Wiens R.D., Zarnegar S., Byers S.L., Chaitman B.R. (1993). Effects of carbon monoxide exposure in patients with documented cardiac arrhythmias. J. Am. Coll. Cardiol..

[10] Ozyurt A., Karpuz D., Yucel A., Tosun M.D., Kibar A.D., Hallioglu O. (2017). Effects of Acute Carbon Monoxide Poisoning on ECG and Echocardiographic Parameters in Children. Cardiovasc. Toxicol..

[11] Atescelik M., Bozdemir M.N., Yildiz M., Gurbuz S., Ayranci M., Goktekin M.C., Kobat M.A., Dagli M.N., Eken C. (2012). QT dispersion in carbon monoxide poisoning. Eur. Rev. Med. Pharmacol. Sci..

[12] Temrel T.A., Bilge S. (2020). Myocardial Repolarization Parameters and Neutrophil-to-Lymphocyte Ratio are Associated with Cardiotoxicity in Carbon Monoxide Poisoning. Cardiovasc. Toxicol..

[13] Gürkan Y., Canatay H., Toprak A., Ural E., Toker K. (2002). Carbon monoxide poisoning—A cause of increased QT dispersion. Acta Anaesthesiol. Scand..

[14] Cho D.H., Ko S.M., Son J.W., Park E.J., Cha Y.S. (2021). Myocardial Injury and Fibrosis from Acute Carbon Monoxide Poisoning: A Prospective Observational Study. JACC Cardiovasc. Imaging.

[15] Pietrasik G., Zaręba W. (2012). QRS fragmentation: Diagnostic and prognostic significance. Cardiol. J..

[16] Kanjanahattakij N., Rattanawong P., Riangwiwat T., Prasitlumkum N., Limpruttidham N., Chongsathidkiet P., Vutthikraivit W., Crossey E. (2018). Fragmented QRS and mortality in patients undergoing percutaneous intervention for ST-elevation myocardial infarction: Systematic review and meta-analysis. Ann. Noninvasive Electrocardiol..

[17] Qaddoura A., Digby G.C., Kabali C., Kukla P., Tse G., Glover B., Baranchuk A.M. (2018). Use of fragmented QRS in prognosticating clinical deterioration and mortality in pulmonary embolism: A meta-analysis. Ann. Noninvasive Electrocardiol..

[18] Altunova M., Püşüroğlu H., Karakayalı M., Sahin A.A., Demir A.R., Yilmaz E., Cizgici A.Y., Erturk M. (2022). Relationship Between Fragmented QRS Complex and Long-Term Cardiovascular Outcome in Patients with Essential Hypertension. Anatol. J. Cardiol..

[19] Saraçoğlu E., Vuruşkan E., Kılıç S., Cekici Y., Onur B., Arslan Y., Kilic E., Aykut O. (2018). Predicting Cardiotoxic Effects of Carbon Monoxide Poisoning Using Speckle Tracking Echocardiography. Cardiovasc. Toxicol..

[20] Das M.K., Khan B., Jacob S., Kumar A., Mahenthiran J. (2006). Significance of a fragmented QRS complex versus a Q wave in patients with coronary artery disease. Circulation.

[21] Raub J.A., Mathieu-Nolf M., Hampson N.B., Thom S.R. (2000). Carbon monoxide poisoning—A public health perspective. Toxicology.

[22] Marius-Nunez A.L. (1990). Myocardial infarction with normal coronary arteries after acute exposure to carbon monoxide. Chest.

[23] Li B., Gao X., Wang W., Zhu B., Qiao Q. (2022). Effect of early intervention on short-term prognosis of patients with myocardial injury induced by acute carbon monoxide poisoning. ESC Heart Fail..

[24] Henry C.R., Satran D., Lindgren B., Adkinson C., Nicholson C.I., Henry T.D. (2006). Myocardial injury and long-term mortality following moderate to severe carbon monoxide poisoning. JAMA.

[25] Satran D., Henry C.R., Adkinson C., Nicholson C.I., Bracha Y., Henry T.D. (2005). Cardiovascular manifestations of moderate to severe carbon monoxide poisoning. J. Am. Coll. Cardiol..

[26] Basaran Y., Tigen K., Karaahmet T., Isiklar I., Cevik C., Gurel E., Dundar C., Pala S. (2011). Fragmented QRS complexes are associated with cardiac fibrosis and significant intraventricular systolic dyssynchrony in nonischemic dilated cardiomyopathy patients with a narrow QRS interval. Echocardiography.

[27] Guo R., Zhang J., Li Y., Xu Y., Tang K., Li W. (2012). Prognostic significance of fragmented QRS in patients with non-ST elevation myocardial infarction: Results of a 1-year, single-center follow-up. Herz.

[28] Sha J., Zhang S., Tang M., Chen K., Zao X., Wang F. (2011). Fragmented QRS is associated with all-cause mortality and ventricular arrhythmias in patient with idiopathic dilated cardiomyopathy. Ann. Noninvasive Electrocardiol..

[29] Das M.K., Michael M.A., Suradi H., Peng J., Sinha A., Shen C., Mahenthiran J., Kovacs R.J. (2009). Usefulness of fragmented QRS on a 12-lead electrocardiogram in acute coronary syndrome for predicting mortality. Am. J. Cardiol..

[30] Tanriverdi Z., Dursun H., Simsek M.A., Unal B., Kozan O., Kaya D. (2015). The Predictive Value of Fragmented QRS and QRS Distortion for High-Risk Patients with STEMI and for the Reperfusion Success. Ann. Noninvasive Electrocardiol..

[31] Erdem F.H., Tavil Y., Yazici H., Aygul N., Abaci A., Boyaci B. (2013). Association of fragmented QRS complex with myocardial reperfusion in acute ST-elevated myocardial infarction. Ann. Noninvasive Electrocardiol..

[32] Kocaman S.A., Cetin M., Kiris T., Erdogan T., Canga A., Durakoglugil E., Satiroglu O., Sahinarslan A., Cicek Y., Sahin I. (2012). The importance of fragmented QRS complexes in prediction of myocardial infarction and reperfusion parameters in patients undergoing primary percutaneous coronary intervention. Turk. Kardiyol. Dern. Ars..

[33] Tanriverdi Z., Dursun H., Kaya D. (2016). The Importance of the Number of Leads with fQRS for Predicting In-Hospital Mortality in Acute STEMI Patients Treated with Primary PCI. Ann. Noninvasive Electrocardiol..

[34] Yıldırım E., Karaçimen D., Ozcan K.S., Osmonov D., Turkkan C., Altay S., Ceylan U.S., Ugur M., Bozbay M., Erdinler I. (2014). The relationship between fragmentation on electrocardiography and in-hospital prognosis of patients with acute myocardial infarction. Med. Sci. Monit..

[35] Torigoe K., Tamura A., Kawano Y., Shinozaki K., Kotoku M., Kadota J. (2012). The number of leads with fragmented QRS is independently associated with cardiac death or hospitalization for heart failure in patients with prior myocardial infarction. J. Cardiol..

[36] Chatterjee S., Changawala N. (2010). Fragmented QRS complex: A novel marker of cardiovascular disease. Clin. Cardiol..

[37] Maehara K., Kokubun T., Awano N., Taira K., Ono M., Furukawa T., Shimizu Y., Maruyama Y. (1999). Detection of abnormal high-frequency components in the QRS complex by the wavelet transform in patients with idiopathic dilated cardiomyopathy. Jpn. Circ. J..

[38] Weidemann A., Klanke B., Wagner M., Volk T., Willam C., Wiesener M.S., Eckardt K.U., Warnecke C. (2008). Hypoxia, via stabilization of the hypoxia-inducible factor HIF-1alpha, is a direct and sufficient stimulus for brain-type natriuretic peptide induction. Biochem. J..

[39] Alter P., Rupp H., Rominger M.B., Vollrath A., Czerny F., Figiel J.H., Adams P., Stoll F., Kolse K.J., Maisch B. (2008). B-type natriuretic peptide and wall stress in dilated human heart. Mol. Cell. Biochem..

[40] Davutoglu V., Gunay N., Kocoglu H., Gunay N.E., Yildirim C., Cavdar M., Tarakcioglu M. (2006). Serum levels of NT-ProBNP as an early cardiac marker of carbon monoxide poisoning. Inhal. Toxicol..

[41] Rich M.W. (2006). Epidemiology, clinical features, and prognosis of acute myocardial infarction in the elderly. Am. J. Geriatr. Cardiol..

[42] Robert L. (1999). Aging of the vascular-wall and atherosclerosis. Exp. Gerontol..

[43] Lakatta E.G., Levy D. (2003). Arterial and cardiac aging: Major shareholders in cardiovascular disease enterprises: Part I: Aging arteries: A “set up” for vascular disease. Circulation.

[44] Lakatta E.G., Levy D. (2003). Arterial and cardiac aging: Major shareholders in cardiovascular disease enterprises: Part II: The aging heart in health: Links to heart disease. Circulation.

[45] Abbate R., Prisco D., Rostagno C., Boddi M., Gensini M.F. (1993). Age-related changes in the hemostatic system. Int. J. Clin. Lab. Res..

[46] Matts J.P., Karnegis J.N., Campos C.T., Fitch L.L., Johnson J.W., Buchwald H., POSCH Group (1993). Serum creatinine as an independent predictor of coronary heart disease mortality in normotensive survivors of myocardial infarction. POSCH Group. J. Fam. Pract..

[47] Friedman P.J. (1991). Serum creatinine: An independent predictor of survival after stroke. J. Intern. Med..

[48] Schillaci G., Reboldi G., Verdecchia P. (2001). High-normal serum creatinine concentration is a predictor of cardiovascular risk in essential hypertension. Arch. Intern. Med..

[49] Gibson C.M., Pinto D.S., Murphy S.A., Morrow D.A., Hobbach H.P., Wiviott S.D., Giugliano R.P., Cannon C.P., Antman E.M., Braunwold E. (2003). Association of creatinine and creatinine clearance on presentation in acute myocardial infarction with subsequent mortality. J. Am. Coll. Cardiol..

[50] Yılmaz E., Aydın E., Çamcı S., Aydin E. (2022). Frequency of fragmented QRS on ECG and relationship with left ventricular dysfunction in patients with subclinical hypothyroidism. Eur. Rev. Med. Pharmacol. Sci..

